# Clinical pharmacists in Dutch general practice: an integrated care model to provide optimal pharmaceutical care

**DOI:** 10.1007/s11096-021-01304-4

**Published:** 2021-07-03

**Authors:** Ankie Hazen, Vivianne Sloeserwij, Bart Pouls, Anne Leendertse, Han de Gier, Marcel Bouvy, Niek de Wit, Dorien Zwart

**Affiliations:** 1 Department of General Practice, Julius Center for Health Sciences and Primary Care, University Medical Center Utrecht (UMCU), Utrecht University, Universiteitsweg 100, 3584 CG Utrecht, The Netherlands; 2 Clinical Pharmacist, Health Care Center Maarn, Raadhuislaan 3, 3951 CH Maarn, The Netherlands; 3 Present Address: Research Department Sint Maartenskliniek, Hengstdal 3, 6574 NA Ubbergen, The Netherlands; 4 Department of Pharmacotherapy, -Epidemiology and -Economics, University of Groningen, Antonius Deusinglaan 1, Building 3214, 9713 AV Groningen, The Netherlands; 5 Department of Pharmaceutical Sciences, Utrecht University, Universiteitsweg 99, 3584 CG Utrecht, The Netherlands

**Keywords:** Clinical pharmacy, General practice, Integrated care, Medication safety, Primary care

## Abstract

*Background* Medication-related harm is a major problem in healthcare. New models of integrated care are required to guarantee safe and efficient use of medication. *Aim* To prevent medication-related harm by integrating a clinical pharmacist in the general practice team. This best practice paper provides an overview of 1. the development of this function and the integration process and 2. its impact, measured with quantitative and qualitative analyses. *Setting* Ten general practices in the Netherlands. *Development and implementation of the (pragmatic) experiment* We designed a 15-month workplace-based post-graduate learning program to train pharmacists to become clinical pharmacists integrated in general practice teams. In close collaboration with general practitioners, clinical pharmacists conduct clinical medication reviews (CMRs), hold patient consultations for medication-related problems, carry out quality improvement projects and educate the practice staff. As part of the Pharmacotherapy Optimisation through Integration of a Non-dispensing pharmacist in a primary care Team (POINT) intervention study, ten pharmacists worked full-time in general practices for 15 months and concurrently participated in the training program. Evaluation of this integrated care model included both quantitative and qualitative analyses of the training program, professional identity formation and effectiveness on medication safety. *Evaluation *The integrated care model improved medication safety: less medication-related hospitalisations occurred compared to usual care (rate ratio 0.68 (95% CI: 0.57–0.82)). Essential hereto were the workplace-based training program and full integration in the GP practices: this supported the development of a new professional identity as clinical pharmacist. This new caregiver proved to align well with the general practitioner. *Conclusion *A clinical pharmacist in general practice proves a feasible integrated care model to improve the quality of drug therapy.

## Facilitators of best practice

- Willingness of both GP and clinical pharmacist to collaborate in improving pharmaceutical care.
- Clinical pharmacists providing a broad range of clinical pharmacy services, including face-to-face clinical medication reviews and follow-up, quality improvement projects and team education.
- An elaborate workplace-based education program of small group learning that includes training in consultation skills and clinical reasoning.

## Barriers to best practice

- Scepticism from community pharmacists and GPs about this new pharmacist role and hesitation among GPs to delegate activities to the clinical pharmacist. Overcome by demonstrating the additional value of this new concept through rigorous scientific research, setting up a postgraduate training program and sharing successes.
- Transitioning from community pharmacist to having a more clinically oriented role as clinical pharmacist in general practice. Overcome by peer support, mentoring and GP supervision to enhance role progression and professional identity formation.
- Funding. Overcome by utilising health care innovation funds and negotiating joint contracts between general practice and community pharmacy.

## Background

With the ageing of the population, the increasing number of patients with multimorbidity and polypharmacy add to the complexity of pharmacotherapy. To address this complexity, it is key to maintain an adequate picture of patient needs. General practitioners (GPs) and community pharmacists commonly have longstanding relationships with their patients being with them for greater parts of their lives. Where GPs have a relatively complete overview of the patient’s clinical status and act as ‘gatekeeper’ to secondary care; the community pharmacist has a complete overview of the patient’s medication. In other words, to provide optimal pharmaceutical care GPs and pharmacists should work closely together.

The current care model in primary care does not facilitate cooperation between GPs and community pharmacists. To give two examples: GPs and pharmacists each have their own patient record leading to scattered information and visits to GPs and pharmacists lack synchronisation making it hard to find time for communal patient consultation. Pharmacists’ primary responsibility is dispensing which leads to underutilisation of their knowledge and skills [1].

We postulate that integration of a clinical pharmacist in the primary care team can overcome these barriers. This model allows pharmacists to take integral responsibility for the quality of the patient’s pharmacotherapy. The GP and pharmacist can complement each other and share the responsibility over the patient’s pharmacotherapy to provide optimal care for the individual patient. Yet, it requires a paradigm shift to transition the pharmacist from the pharmacy to the general practice.

This paradigm shift has commenced in some settings in Canada, the United States of America (USA), New-Zealand and the United Kingdom (UK) where pharmacists operate within the general practice with promising results so far [2, 3]. In the Netherlands, this care model recently has been tested in the POINT study (Pharmacotherapy Optimisation through Integration of a Non-dispensing pharmacist in a primary care Team) [4]. This care model differs to some extent to the currently known models:

1. The development and implementation are driven by pharmacists and GPs (i.e. not centrally or government-driven);
2. It includes a 15-month, extensive workplace-based education program based on experiential learning;
3. The clinical pharmacists are generalists that provide all-inclusive services: adopted to and available for all patients of the general practice.

This best practice paper (1) provides details on the development and implementation of this integrated care model and (2) gives a short overview of the results on improving safe and effective pharmaceutical care. Herewith, the paper aims to provide ideas and tools to practitioners and policymakers who intent to introduce a similar care model.

### Aim

We aimed to improve the quality of pharmacotherapy by integrating a clinical pharmacist in the general practice team to provide effective pharmaceutical care in close collaboration with the GP.

### Objectives

1. Develop, implement and evaluate a post-graduate training program for clinical pharmacists in general practice.
2. Evaluate the role development of clinical pharmacists in general practice.
3. Determine effectiveness and acceptability of the integrated care model.

### Development of the function of clinical pharmacist in general practice

In 2014, a multidisciplinary team of clinical, social and educational researchers, GPs and clinical pharmacists designed an integrated care model in which pharmacists are a member of the multidisciplinary general practice team. To enable pharmacists to take on their new role in this model, a 15-month training program was developed [5]. Both care model and training program were implemented and evaluated in the POINT study.

The fundament of this pharmaceutical care model consisted of three components: an extensive training program, operating from a single general practice and a broad job description. The components are explained in more detail below.

#### A 15-month training program for pharmacists on integrated pharmaceutical care provision

The main emphasis of the training program was on patient-centred care: involving patients and identifying their needs. The education framework was based on workplace and experiential learning with learning competencies and learning outcomes formulated from the Canmeds framework. Inspiration was drawn from the academic clinical pharmacy training courses in the USA, Canada and the UK. The education program was further developed according to the “The Ten Steps” of van Merriënboer et al. which supports complex learning [6]. Development and execution of the program was in close partnership with the university department for general practice specialty training.

**Fig. 1:**
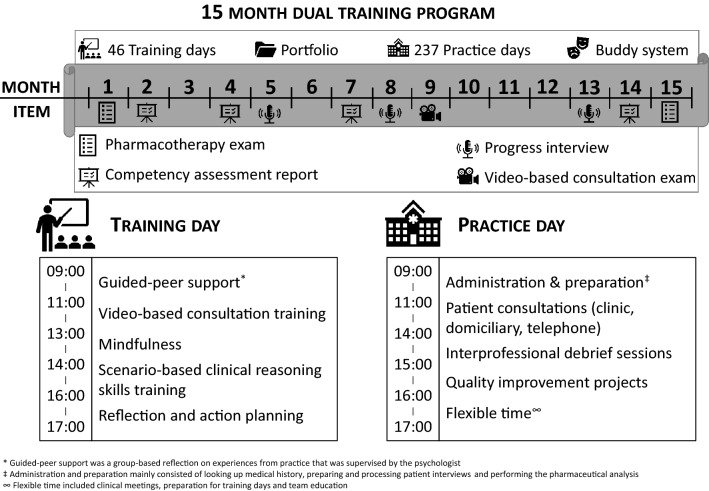
overview of the 15 month dual training program for clinical pharmacists in general practice

The 15-month training program consisted of 46 study days that were scheduled between practice days (see Fig. 1). The program aimed to shift the pharmacists’ focus from product-oriented to patient-centred care. To help the pharmacists make the transition to patient-centred care the training program included consultation skills training and clinical reasoning classes. During the program, the pharmacists learned how to adjust pharmacotherapy fitted to the individual patient, to voice their concerns, beliefs and expectations regarding the medication as advised by the GP and to take the corresponding responsibility. Inherent to this responsibility is uncertainty on the consequences which the pharmacists also had to learn to deal with. Specific training and workplace activities were developed accordingly. The study days were supervised by an experienced clinical pharmacist and a psychologist specialised in education of patient communication skills in health care. Further support for professional role development was provided through mentorship and a buddy program.

#### Clinical pharmacists operate in a single general practice team

The integration of a clinical pharmacist is a dynamic and often time-consuming process and therefore we chose for the pharmacists to work in a single (large) practice rather than across multiple practices, like in the UK. We expected that this would more likely lead to speedy integration. Being only in one practice allowed for everyday work interactions, including joint patient consultations, formal debrief sessions to discuss patients’ treatment plans and frequent encounters to discuss ad hoc patient queries. To further enhance integration, each pharmacist received clinical supervision from at least one GP, joined and/or organised multidisciplinary clinical team meetings and liaised with the wider multidisciplinary team such as community pharmacists and nurse practitioners to streamline care processes.

#### Job description of the clinical pharmacist in general practice

The job description of the clinical pharmacist consists of the full spectrum of clinical pharmacy services: patient-centred care, quality improvement and education of health care providers. This broad set of clinical services was realised in three phases. First, literature was studied on pharmaceutical care in practice and on barriers of pharmaceutical care implementation in primary care. Next, the services were piloted by a clinical pharmacist in two general practice settings and experiences from clinical pharmacist in the UK were collected in interviews. Finally, the set of services were presented to patients in interviews and focus groups and finalised.

Clinical pharmacy services consisted of face-to-face clinical medication reviews including follow-up, medication reconciliation for patients discharged from the hospital and consultations about specific drug-therapy problems. To gain the patient’s trust and have the most complete information about the patient’s situation reviews were preferably performed at the patient’s home. Quality of care services consisted of quality improvement projects on care processes and prescribing (e.g. optimising the repeat prescription process, reducing inappropriate use of benzodiazepines) tailored to the patient population of the general practice. Training in pharmacotherapy was provided for all members of the general practice team as well as at regional pharmacotherapy meetings.

### Challenges in the development of the pharmaceutical care model

The key challenge in developing the pharmaceutical care model was the initial reserve from stakeholders such as the national professional organisation of pharmacists (KNMP), local community pharmacists and GPs about this new clinical role for pharmacists. This did not come as a surprise as previous research already indicated that introducing a clinical pharmacist in general practice can lead to debate, as new roles put professional boundaries under pressure [7]. We carried out a mixed-method study to map the controversy and consensus amongst stakeholders on this topic to get a better understanding of potential facilitators and barriers [8]. We tried to overcome this challenge by pro-actively keeping stakeholders informed and involved by sharing the benefits of the new care model. Also, the clinical pharmacists liaised with the community pharmacists in the catchment area and quality projects were done collaboratively.

### Implementation of the (pragmatic) intervention

Almost 90 community and hospital pharmacists with varying levels of experience applied for the training program and the position as clinical pharmacist in general practice. Of those applicants ten pharmacists were recruited. General practices from the Julius GP Network (University Medical Centre Utrecht), healthcare network Almere (Zorggroep Almere) and the Registration Network of GPs Associated with Leiden University (RNUH-LEO) were pro-actively invited to host the clinical pharmacist-trainees and ten were selected. These practices (that were all part of a multidisciplinary health care centre) were willing to participate in the project and to contribute to role development and evaluation of the new clinical pharmacists’ role. The main hurdles in recruiting practices were that practices had either insufficient room to house the pharmacist or had concerns about the unknown implications of participating in and contributing to this new care model. For example, the uncertainty on what happened after the clinical pharmacist had ended the training program. Every clinical pharmacist-trainee was matched to a general practice and worked exclusively in that practice for 15 months (March 2014-June 2015).

To allow for a smooth start and to aid integration, the clinical pharmacists’ services were tailored to the general practice needs. At the start of the intervention priorities for the practice were agreed and focus areas for the clinical pharmacist were decided. Overall, the main target population for the clinical pharmacist were the elderly with multimorbidity and polypharmacy as they are at the highest risk of experiencing drug-related problems. Priorities and practice needs were reviewed on a regular basis and the pharmacist’s role was adapted accordingly.

### Evaluation: does the model work, and if so: how?

This complex intervention was evaluated following the Medical Research Council framework. Hence, both quantitative and qualitative approaches were used to evaluate the integrated model [9]. An overview of evaluations, methods, data collection and outcome measures is provided in Table 1.

**Table 1:** Overview of qualitative and quantitative evaluations, method and data collection and outcome measures

Timeline	Evaluation	Method and/or data collection	Outcomes
At the start of the intervention	Stakeholders perspectives and opinion about new care model	Interviews and surveys using Q-methodology	Consensus and controversy about integrating pharmacists in general practice [8]
Throughout the intervention	Training program	Two-stage interviews, peer feedback and individual reflections from pharmacists	Learning through boundary crossing and professional identity formation of general practice pharmacists [10]
At 3 pre-set dates during intervention	Effectiveness of clinical medication reviews	Observational cross-sectional studies	Drug therapy problems, implementation of recommendations, patient satisfaction [11]
After completion of the intervention	Clinical effectiveness of the intervention	Non-randomised controlled trial	Medication-related hospitalisations, healthcare costs, drug burden index, prescribing indicators [12, 13]
Post-intervention	GPs’ perspectives	Interviews with GPs, evaluated using realist evaluation methodology	Professional identity alignment between GP and clinical pharmacist as working mechanism [14]

### Training program and developing a new role

Of the ten participating pharmacists, nine successfully finished the training program. The training program was perceived as highly valuable to the pharmacist’s role development, according to the participating pharmacists:

I notice that, compared to a year ago, I use consultation skills and apply them to my professional behaviour. As a result, I achieve more. The design of the [training] program ensured that I learned a lot. [5]

The biggest development was [learning] to cope with feeling responsible for the patient. Reflection during the peer feedback sessions was a prerequisite to learning. It’s important to focus on certain processes that you go through, and the feedback sessions forced you to put them into words. [10]

Analysis of data derived from two-stage interviews, reports of weekly peer feedback session and individual reflective accounts on competency development collected during and after the training program demonstrated that reflective learning both during formal training days and at the workplace helped the pharmacist to develop a patient-centred professional identity. The pharmacists gradually developed a professional identity that differed substantially from their previous roles. Our study showed that the transformation of pharmacists in general practice expanded the traditional ‘being a medication expert’ with being a clinician, being a professional who takes responsibility for the patient’s pharmacotherapy based on the patient’s needs, being an anticipator by proactively detecting medication safety issues and being a collaborator by bridging care between community pharmacy and general practice. We also noticed remarkably little resistance and tensions about role and responsibilities between community pharmacists and the clinical pharmacist. Their roles can be complementary.

The pharmacists described a fundamental change in their thinking and acting and developed a new vision that was then embedded in their practice:

The fact that I am a real part of the team, that I know how to talk with patients and how to coach them, that I’ve found a working mode for general practice. [10]

### Integration in general practice

From interviews with GPs we learned that another process was essential for this care model to be successful: alignment of professional identities between GP and pharmacist. For GPs, this entailed valuing the different working approaches of the pharmacists compared to their own, and, over time, starting to change opinions about sharing responsibility in pharmaceutical care. The alignment of professional identities took place during ‘coffee corner and corridor talking’ and during short daily debrief sessions. These interactions occur less frequently when GPs and community pharmacists work in their separate practices (even if this is in the same building) and identity alignment does not happen.

### Impact on improving the quality of pharmaceutical care

We determined the clinical effectiveness of this new care model by measuring medication-related hospitalisations. We collected data on all acute, unplanned hospitalisations between 2013 (baseline period) and 2015 (intervention period) of intervention and control practices. We performed a multi-step case‐by‐case assessment of all acute admissions, based on a validated algorithm to identify medication‐related hospitalisations. A total of 11 928 high‐risk patients were included in the analysis and 822 medication‐related hospitalisations were identified. In general practices with fully integrated clinical pharmacists, the rate of medication related hospitalisations among high‐risk patients was lower compared to usual care (rate ratio 0.68 (95% confidence interval 0.57–0.82)) [12].

In the clinical medication reviews carried out by the clinical pharmacists a median of 5 drug therapy problems were identified. The pharmacists provided therapy recommendations of which 83% were implemented. In 78%, the implementation of the recommendations actually solved the drug therapy problem [11]. These numbers are high compared to other models of pharmacist-led services. This was also reflected by less patients reporting to experience side-effects after a CMR by the clinical pharmacist compared to patients receiving no such CMR [14].

We also looked at drug burden index, direct healthcare costs and the quality of prescribing (measured via prescribing quality indicators) but here no differences were found with usual care [12, 13].

## Discussion

In this best practice paper, we described how we developed, implemented and evaluated the integration of a clinical pharmacist in the Dutch general practice team. Ten general practices integrated a clinical pharmacist in training in their team aiming for full provision of integrated pharmaceutical care. The 15-month post-graduate workplace-based training program supported the professional transition to patient-centred, clinical pharmaceutical care practitioner. We demonstrated that this model improves the quality and safety of pharmaceutical care, e.g., by the positive impact on medicines-related hospitalisations, by solving drug therapy problems and by patients experiencing less side effects.

When the funded program ended in 2015, five pharmacists were employed either directly by the general practice or in liaison with the community pharmacy. These clinical pharmacists are still working in general practice today (April 2021). In addition to this at least two other Dutch general practices have employed a clinical pharmacist. This highlights the sustainability and acceptability of the integrated care model, which is also reflected by the implementation of similar models in other countries [15–17].

We believe that three elements are key to make this model successful. Training is the first key element. So far, training programs for clinical pharmacists in general practice that were developed in other countries showed considerable variety [18, 19]. It is a significant transition from community-based medication-focussed care to person-centred care in shared responsibility with the GP. Pharmacists are traditionally trained to eliminate error and they are not familiar with proactively taking responsibility for the patient’s pharmacotherapy and the decision-making process in general practice that involves weighing up risks and benefits. A post-graduate training program for clinical pharmacists in general practice needs a curriculum that focusses on patients’ needs instead of medicines and should include both training in consultation and clinical reasoning skills and peer support and supervision from experienced pharmacists and GPs to enhance integration, role progression and skill mix between GP and pharmacist. In England, a 18-month training program for general practice pharmacists that incorporates these aspects, including shared decision making, is broadly implemented [20].

The second key element is full integration of the clinical pharmacist into the general practice team to enhance identity alignment. Jorgenson et al. published guidelines with evidence-based recommendations for successful integration of clinical pharmacists, and we consider most of these to contribute to identity alignment [21]. Although pharmacist and GP share the same goal–providing the best possible care for patients–they do not necessarily make use of each other’s areas of expertise. This lack of shared professional perspective starts at university where interprofessional education is limited and works its way into practice where a lack of interprofessional communication can lead to working in silos. The integrated care model described in this paper shows that working in the same team and in the same location is an enabling factor to improve collaboration and to build trust, which has previously been recognised [22]. We believe that working at the same ‘clinical floor’ stimulates all aspects of integration, both formally (eg. same usage of and knowledge of guidelines and medical records) and informally (eg. speaking the same language, using similar frames of reference and building trust). In all these moments, professional identities are explored and identity alignment between GP and pharmacist can take place.

The third key element is that both pharmacists and GPs acknowledge a need for change of the current pharmaceutical care, and moreover, are willing to improve its quality. Worldwide, the need for change of the current pharmaceutical care model is increasingly acknowledged amongst GPs [23]. Still, the integration of the clinical pharmacist in the general practice team is a process that takes time and pharmacists and GPs must actively engage to make the model work. Clinical pharmacy services need to be tailored to the practice needs and consequently the pharmacist’s roles and responsibilities can change. In our model, the clinical pharmacist worked under supervision of the GP. Time will tell whether the level of responsibility and accountability of the clinical pharmacist further develops. In the UK there are examples where clinical pharmacists have become partner in the GP surgery. It is open for debate whether obtaining independent prescribing authority– something that is not yet possible in the Netherlands–would enhance this process.

Several barriers to best practice have been identified. In the process of full integration, the pharmacists had to overcome two barriers: 1. scepticism about this new role for pharmacists and 2. the professional transition of becoming a clinical care provider in general practice. Although scepticism was perceived nationally [8], it was mainly experienced and overcome locally. Where some community pharmacists were defensive when the pharmacists started, they quickly experienced the advantages of having a colleague based at the general practice. This resonates with the design of this integrated care model: not to eliminate but to complement the current medication focussed model. A last –and remaining– barrier, is funding.

Compared to similar integrated care models in other counties, the model described in this paper is a bottom-up approach created by a multidisciplinary team and funded by both national research funding bodies as well as funding by an innovative healthcare insurer. The main advantage of this approach is that all parties involved are willing to innovate, which has led to close collaboration and full integration of the pharmacists. In other words, the participating practices in our study were frontrunners, potentially not reflecting average practice. Hence, broad and successful implementation of this model requires a full-blown implementation plan and a sustainable funding model.

## Conclusion

A clinical pharmacist integrated in general practice proves an example of integrated care that serves to improve quality and safety of pharmaceutical care. Broader implementation is recommended and should be based on the following three cornerstones: full integration of the pharmacist into the general practice team allowing for shared responsibility between pharmacist and GP and identity alignment, providing additional education for pharmacists to become a clinical care provider and delivering a broad range of clinical pharmacy services tailored to the needs of the general practice population.

## Data Availability

All data that has been used for this best practice article stems for previously published articles on the POINT project. Please see the reference list for the relevant articles.
